# Sprint Kayaking Performance Enhancement by Isometric Strength Training Inclusion: A Randomized Controlled Trial

**DOI:** 10.3390/sports9020016

**Published:** 2021-01-21

**Authors:** Danny Lum, Tiago M. Barbosa, Govindasamy Balasekaran

**Affiliations:** 1 Sport Science and Sport Medicine, Singapore Sport Institute, Singapore 397630, Singapore; 2 Physical Education and Sports Science, National Institute of Education, Nanyang Technological University, Singapore 637616, Singapore; barbosa@ipb.pt (T.M.B.); govindasamy.b@nie.edu.sg (G.B.); 3 Polytechnic Institute of Braganca, 5300 Braganca, Portugal; 4 Research Centre in Sports, Health and Human Development (CIDESD), 5001 Vila Real, Portugal

**Keywords:** peak force, rate of force development, isometric squat, isometric bench press, isometric prone bench pull

## Abstract

Performing isometric strength training (IST) can enhance various sports performance. This study compared the effects of including IST on sprint kayaking performance as compared to traditional strength training. Twenty sprint kayaking athletes (age 22 ± 4 year, stature 1.71 ± 0.09 m, body mass 72.0 ± 11.4 kg) performed a 200-m kayak ergometer time trial (200mTT), isometric squat (IsoSqT), isometric bench press (IsoPress) and isometric prone bench pull (IsoPull) during the pre- and post-tests. Athletes were randomly assigned to either traditional strength training (TRAD) or IST group. Both groups performed a similar strength training program twice a week for six weeks. However, half the volume for squat, bench press and prone bench pull were replaced by IsoSqT, IsoPress and IsoPull, respectively, for the IST group. IsoSqT was performed at 90° knee angle, while IsoPress and IsoPull were performed at 90° and 120° elbow angles, respectively. Each isometric contraction was performed with maximum intensity and sustained for three seconds. A significant main time effect was observed for 200mTT (*p* < 0.001, ƞ^2^_p_ = 0.68) and all isometric strength measures (*p* = 0.001–0.032, ƞ^2^_p_ = 0.24–0.76) except rate of force development at 0–90 ms (RFD90) obtained from IsoSqT120 and IsoPress90. A group main effect was observed in RFD90 obtained from IsoSqT120 and IsoPull120 (*p* = 0.003–0.004, ƞ^2^_p_ = 0.37–0.39). Time x Group interaction was observed for 200mTT (*p* = 0.027, ƞ^2^_p_ = 0.68), peak force obtained from IsoSqT90, IsoPress90, and IsoPull120 (*p* = 0.004–0.006, ƞ^2^_p_ = 0.36–0.38) and RFD90 obtained from IsoSqT120 and IsoPull120 (*p* = 0.012–0.015, ƞ^2^_p_ = 0.28–0.30). Inclusion of IST resulted in greater improvement for sprint kayaking and strength performances then TRAD alone.

## 1. Introduction

Strength training is an integral component in the physical preparation of sprint kayakers, and improvement in muscular strength has been associated with improved kayaking performance [1,2,3]. For example, McKean and Burkett [3] reported that a 6.5–13% increase in 1 repetition maximum bench press and a 2.3–10% increase in 1 repetition maximum pull up coincided with improvement of 1% in kayaking time. Several studies have also reported that force production of the lower limb is integral to kayaking performance [4,5,6]. For example. Nilsson and Rosdahl [6] reported that lower limb force contributed to about 21% of mean paddle stroke force and 16% of mean kayak speed. However, no study has reported the effects of lower limb strength training on kayaking performance. It would be of interest to know if strength training for the lower limb would further enhance kayaking performance.

To the authors’ knowledge, there is only one study available in the literature that conducted a randomized controlled trial to investigate the effects of different strength training methods on kayaking performance [2]. Liow and Hopkins [2] reported that bench press and bilateral dumbbell prone lifts performed with a slow concentric phase resulted in greater improvement in the initial acceleration phase as compared to performing the concentric phase rapidly. Despite this finding, a study that compares the effects of different modes of strength training with the traditional strength training (TRAD) method, which involves concentric and eccentric muscle action while lifting heavy weights (e.g., bench press and bench pull), is lacking.

Isometric strength training (IST) is a mode of strength training that involves the production of force by the skeletal muscles without any external movement [7,8,9,10,11,12]. This mode of strength training has been compared with isoinertial, isokinetic and plyometric training [7,8,9,13,14,15,16]. It was reported that IST has several advantages over the dynamic mode of strength training, and these include lower energy cost, greater improvement in tendon stiffness and joint angle-specific strength [8,9,10,12,14]. In addition, Kordi et al. [13] reported greater improvement in track cycling when IST was included as compared to traditional strength training alone. Similar to cycling, the stretch-shortening cycle has minimal contribution to force production during kayaking stroke due to the lack of eccentric contraction [2]. As such, a mode of strength training that enhances cycling performance might also have a positive effect on kayaking performance. To date, no study has investigated the effects of IST on kayaking performance. Furthermore, there is a paucity of randomized controlled trials conducted to investigate the effects of strength training on kayaking performance.

Isometric strength measures of various muscles have resulted in significant correlation to on-water sprint kayaking time and kayak ergometer mean power [5,17,18]. For example, Lum and Aziz [5] reported significant correlation between isometric strength measures obtained from isometric squat (IsoSqT), isometric prone bench pull (IsoPull), and isometric bench press (IsoPress) with 200-m on-water kayaking time and ergometer kayaking mean power, (−0.44 ≤ *r* ≤ −0.88). Furthermore, predicted 200-m on-water kayaking time using regression equation based on peak force (PF) obtained from IsoSqT, IsoPress and IsoPull resulted in only 1.3% standard error from actual 200-m on-water kayaking time. These findings showed that IsoSqT, IsoPress and IsoPull are suitable strength assessments for monitoring sprint kayaking athletes’ strength as peak force obtained from these assessments may help to predict athletes’ sprint kayaking performance.

Although the correlation does not indicate a causation, there is a possibility that if IST were to be performed using IsoSqT, IsoPress and IsoPull, the increment of maximum force production at those joint positions may enhance athletes’ ability to overcome the drag force during each paddling stroke to move the boat faster. Therefore, the aim of the current study was to compare the effects of including IST on kayaking performance as compared to TRAD. As IST was performed at joint positions similar to that adopted during the initiation of the pull phase of kayak stroke, it would most likely result in greater strength increment in those positions than TRAD [10,12,13]—thus, further enhance the ability to overcome drag force during kayaking. Thus, it was hypothesized that performing IST would result in greater improvement to sprint kayaking performance than TRAD.

## 2. Materials and Methods

### 2.1. Participants

Sample power was computed (G*Power, v.3.1.9.2, University of Kiel, Kiel, Germany) assuming as inputs an expected large effect size (for instance, f = 0.4), 5% of error probability for 95% of power, two groups (i.e., 2 conditions), two measurements (i.e., pre- and post-test), correlation among repeated measures of 0.5 and non-sphericity correction of 1. Computation showed that a sample size of at least *n* = 16 was required to obtain a statistical power of 0.85.

Twenty (TRAD—8 male and 2 female, IST—8 male and 2 female) national and collegiate kayak athletes (TRAD—age 21 ± 4 year, stature 1.72 ± 0.10 m, body mass 75.1 ± 11.0 kg, IST—age 22 ± 4 year, stature 1.73 ± 0.05 m, body mass 76.1 ± 8.4 kg) were recruited for this study. These athletes had been performing resistance training at least twice a week and participating in kayaking competitively for at least 2 years; had been training for at least six hours per week for the last 6 months; did not sustain any injury for the last 6 months; and had been training regularly with a kayak ergometer.

Prior to participation, all participants were briefed on the requirements and risks involved with the study. Participants were required to sign a written informed consent prior to the initial testing session. Parental consent was sought for those under the age of 21 years old. The study was ethically cleared by the Nanyang Technological University and Singapore Sport Institute’s Institutional Review Board.

### 2.2. Experimental Approach to the Problem

**Figure 1:**
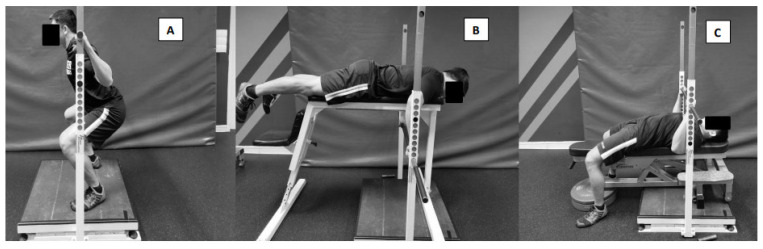
Testing set up for (**A**) isometric squat, (**B**) isometric prone bench pull, (**C**) isometric bench press.

A randomized controlled trial research design was selected. Participants attended a familiarization session 48–72 h prior to the pre-test session to be familiar with the isometric strength tests and to perform the 200-m ergometer kayaking time trial (200mTT) for reliability test analysis. The pre- and post-testing sessions included three maximum isometric strength tests, IsoSqT, IsoPull and IsoPress (Figure 1), followed by 200mTT. Participants were matched for 200mTT mean power before being randomly assigned to either the TRAD or IST group. Participants attended 2 training sessions per week for 6 weeks, each separated by 48–72 h.

### 2.3. Testing Sessions

All pre- and post-tests (72 h upon completion of last training session) were conducted prior to and after the intervention training. Each testing session began with a 5 min moderate-intensity paddle on the kayaking ergometer (Kayakpro Speedstroke Hi Res, Miami Beach, FL, USA), followed by dynamic stretches for upper and lower limb. One minute of recovery period was given prior to commencing the maximum isometric strength tests. Participants performed the IsoSqT, IsoPress and IsoPull tests in a randomized sequence, with a 5 min recovery period in between each test [5]. Upon the completion of the final isometric strength test, they were given a recovery period of 10 min prior to performing the 200mTT [5].

The IsoSqT, IsoPress and IsoPull were performed on the FT700 Isotronic Ballistic Measurement System (Fitness Technology, Adelaide, Australia), incorporating the 400 series force plate (sampling at 600 Hz). A sampling frequency of ≥500 Hz has previously been reported to provide accurate and reliable measurements of peak force, time-specific force values, and rate of force development (RFD) at pre-determined time bands during the IMTP [19]. The procedure of the tests follows that which was previously described by Lum and Aziz [5]. IsoSqT was performed at knee flexion angles of 90° (IsoSqT90) and 120° (IsoSqT120) (full knee extension being 180°); IsoPress was performed at 90° (IsoPress90) and 120° (IsoPress120) elbow flexion angles; and IsoPull was performed at 90° (IsoPull90) and 120° (IsoPull120) elbow angles. Participants performed the tests at each joint angle twice separated by a 2 min recovery period between attempts and a 5 min recovery period between tests. Peak force and rate of force development at 0–90 ms (RFD90) were recorded for all isometric tests. The initiation of contraction was identified as the time corresponding to a force of 20 N above baseline [20].

After completing the isometric strength tests, a 10 min passive rest ensued. Subsequently, athletes performed a 10 min self-paced warm up on the same kayak ergometer. After 2 min of passive recovery, they were instructed to complete a 200 m distance in the shortest time possible. The mean power attained during the 200mTT was used as the criterion measure of kayak performance with a higher mean power indicating a better performance [5]. The 200mTT was selected as a criterion measure to eliminate the effects of weather conditions. Previous study by Lum and Aziz [5] reported very strong correlation between 200mTT mean power and 200-m on-water sprint time (*r* = −0.90).

### 2.4. Training Program

Participants attended 2 training sessions per week for 6 weeks, each separated by 48–72 h. Both TRAD and IST group performed similar strength training program (Table 1). However, for IST, two sets of dynamic squats, bench press and prone bench pull were replaced by IsoSqT, IsoPress and IsoPull, respectively. The IsoSqT, IsoPress and IsoPull involved participants exerting maximal force against a stationary bar as fast and as hard as they could. Each repetition was held for 3 s with 3 s rest in between repetitions [21]. Participants performed the IsoSqT at similar knee flexion angle (~100°), IsoPress (~90°) and IsoPull (~120°) at similar elbow flexion angles, as they adopted when they were initiating the pull phase during 200mTT. Intensity of all dynamic exercises was set as two repetitions in reserve for each set. In addition, the eccentric phase was performed with a 2 s tempo while the concentric phase was performed with the intent to lift as hard and as fast as possible.

All participants were performing similar training program as TRAD for 4 weeks leading up to the commencement of the study. All participants recruited for the study completed every training session and post-tests (i.e., no dropouts).

### 2.5. Statistical Analysis

All tested variables are expressed by Mean (±1 SD) and 95% confidence interval. Between and within session, test-retest reliability was assessed using two-way mixed intraclass correlation coefficients (ICC) and typical error (TE) for 200mTT and all other measured variables, respectively. ICC values were deemed as highly reliable if *r* ≥ 0.80 [22].

Data were analyzed using mixed ANOVAs (between- × within-participant analysis; 2 training groups × 2 testing times; *p* ≤ 0.05) with Bonferroni’s post hoc comparisons using one between-group factor (TRAD and IST) and one within-group factor (pre-training and post-training). Effect size was computed by partial eta-squared (ƞ^2^_p_) and deemed—without effect if 0 < ƞ^2^_p_ ≤ 0.01; small if 0.01 < ƞ^2^_p_ ≤ 0.06; medium if 0.06 < ƞ^2^_p_ ≤ 0.14 and; strong if ƞ^2^_p_ > 0.14. All assumptions to run ANOVAs were checked beforehand, including normality and sphericity. Degrees of freedom were corrected whenever sphericity’s assumption was violated. An independent T-test was used to determine if there were any between-group differences in percentage change for all variables. Cohen’s d was calculated as standardized effect size for mean comparisons, and deemed as—(i) trivial effect size if 0 ≤ *d* ≤ 0.2; (ii) small effect size if 0.2 < *d* ≤ 0.5 and; (iii) moderate effect size if 0.5 < *d* ≤ 0.8; (iv) large effect size if *d* > 0.8.

Associations between change in mean power and change in all isometric strength measures were determined using Pearson’s product-moment correlation (*p* < 0.05). Correlational indices were set at—(i) small, if 0.1 ≤ *r* ≤ 0.29; (ii) moderate, if 0.3 < *r*≤ 0.49; (iii) large, if 0.5 ≤ *r* ≤ 0.69; (iv) very large, if 0.7 ≤ *r* ≤ 0.89; (v) near perfect, if 0.9 ≤ *r* ≤ 0.99; and (vi) perfect, if *r* = 1.

## 3. Results

### 3.1. Reliability of the Measures

The ICC data for 200mTT and all isometric tests measures showed very high repeatability (Appendix A, Table A1). Test-retest data indicted a typical error of 3.0% for 200mTT and 3.6–8.9% for all isometric strength measures.

### 3.2. Time X Group Interactions

Table 2 displays the results of 200mTT and PF from all isometric tests, while Table 3 displays the results of RFD from all isometric tests. Significant large time x group interactions were observed in 200mTT mean power (*p* = 0.027, ƞ^2^_p_ = 0.24), IsoSqT90 PF (*p* = 0.004, ƞ^2^_p_ = 0.38), IsoSqT120 RFD90 (*p* = 0.015, ƞ^2^_p_ = 0.28), IsoPress90 PF (*p* = 0.004, ƞ^2^_p_ = 0.38), IsoPull120 PF (*p* = 0.006, ƞ^2^_p_ = 0.36) and IsoPull120 RFD90 (*p* = 0.012, ƞ^2^_p_ = 0.30). There was no time x group interactions in other measured variables, though small-to-large standardized effect sizes were observed (0.04 ≤ ƞ^2^_p_ ≤ 0.29).

### 3.3. Time Main and Simple Effects

**Figure 2:**
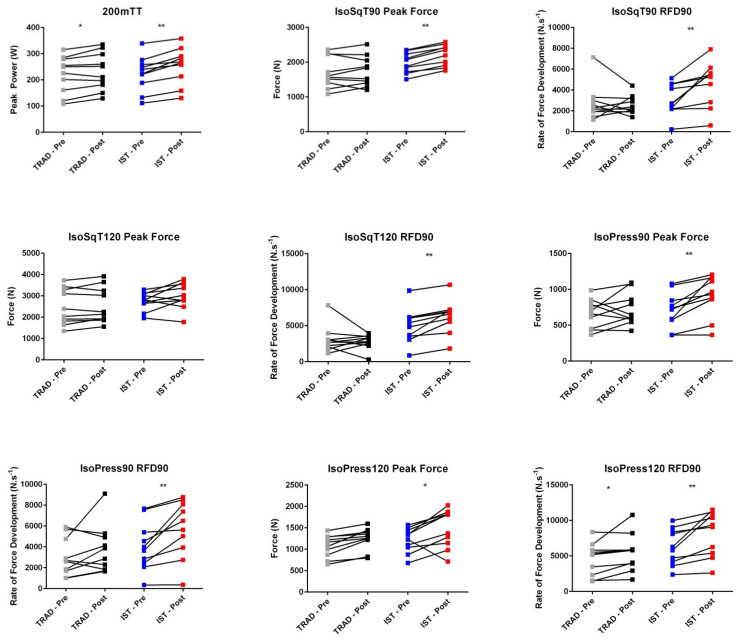

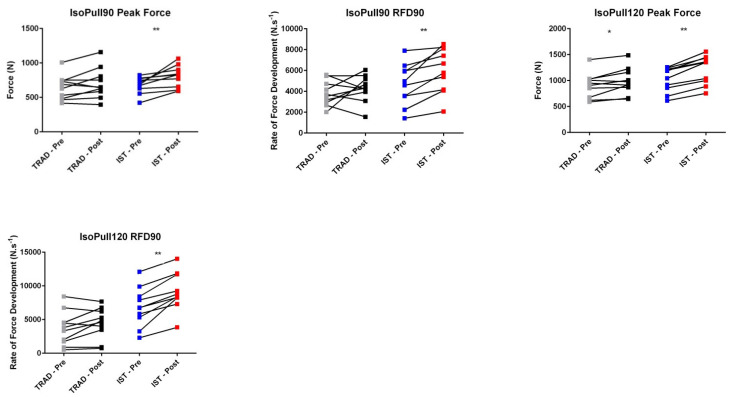
Pre- and post-test measures for 200mTT and all isometric strength variables. * Denotes significant difference (*p* < 0.05). ** Denotes significant difference (*p* < 0.01). Grey cube = TRAD – Pre, black cube = TRAD – Post, blue cube = IST – Pre, red cube = IST – Post.

A significant large main effect for time was observed for 200mTT mean power (*p* < 0.001, ƞ^2^_p_ = 0.68). In addition, a significant large effect for time was observed for all isometric PF (0.0001 ≤ *p* ≤ 0.032, 0.16 ≤ ƞ^2^_p_ ≤ 0.76) and RFD90 (0.001 ≤ *p* ≤ 0.028, 0.24 ≤ ƞ^2^_p_ ≤ 0.65), except RFD90 obtained from IsoSqT120 and IsoPress90. Significant improvement in 200mTT mean power for was observed in both groups (TRAD: *p* = 0.028, *d* = 0.18), IST: *p* < 0.001, *d* = 0.45) (Figure 2). A significant increase in PF and RFD90 obtained from all isometric tests was observed in the IST group (0.001 ≤ *p* ≤ 0.024, 0.57 ≤ *d* ≤ 0.76 and 0.001 ≤ *p* ≤ 0.008, *d* = 0.52, respectively) except for IsoSqT120 PF (*p* = 0.087, *d* = 1.04). However, the only isometric strength measures that were significantly increased in TRAD were IsoPress120 PF (*p* = 0.001, *d* = 0.68), IsoPress120 RFD90 (*p* = 0.048, *d* = 0.38) and IsoPull120 PF (*p* = 0.022, *d* = 0.32).

### 3.4. Group Main Effects

There was no significant group main effect for 200mTT mean power (*p* = 0.695, ƞ^2^_p_ = 0.01). Significant large group main effect was observed for IsoSqT90 PF *(p* = 0.037, ƞ^2^_p_ = 0.22), IsoSqT120 RFD90 (*p* = 0.004, ƞ^2^_p_ = 0.37) and IsoPull120 FD90 (*p* < 0.001, ƞ^2^_p_ = 0.39). No significant group main effect was observed for other isometric strength measures (0.053 ≤ *p* ≤ 0.356, 0.05 ≤ ƞ^2^_p_ ≤ 0.19).

**Figure 3:**
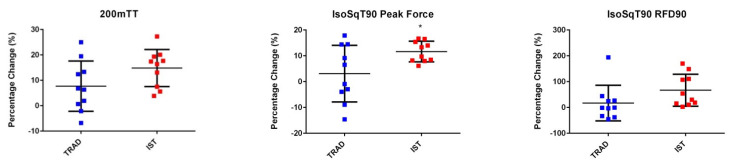

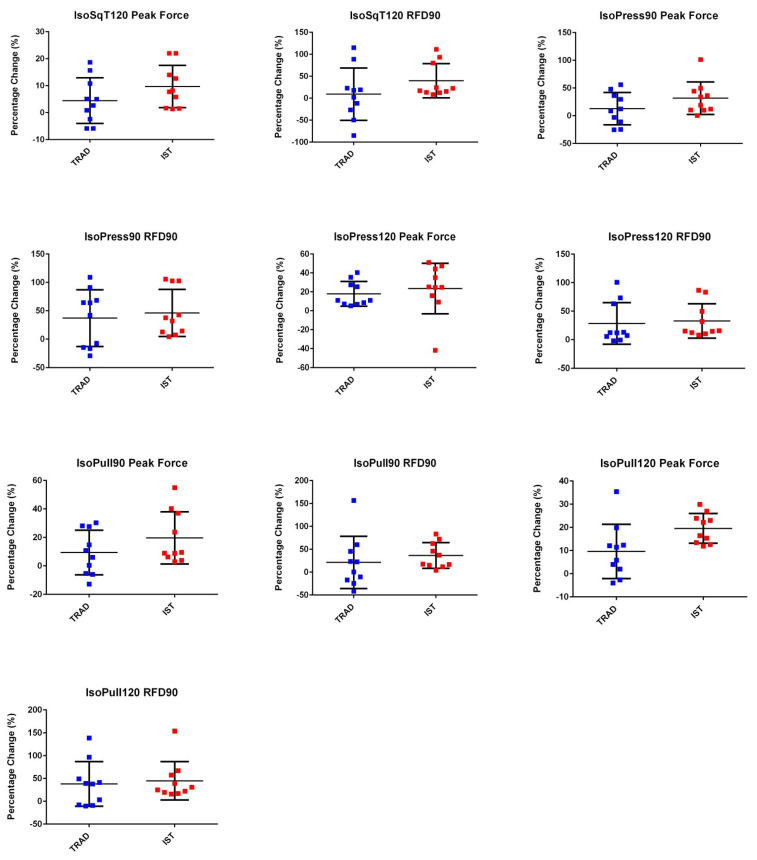
Percentage change for 200mTT and all isometric strength measures. Centre bar represents the mean and error bars represent the standard deviation. * Denotes significant difference (*p* < 0.01). Blue cube = TRAD, red cube = IST.

Figure 3 illustrates the individual changes for all measured variables. Change in IsoSqT90 PF was significantly higher in the IST than the TRAD group (*p* = 0.002, *d* = 1.03). A non-significant difference was observed for other variables. However, compared to TRAD, IST resulted in large effects sizes for changes in 200mTT mean power (*p* = 0.351, *d* = 0.81), IsoSqT120 PF (*p* = 0.17, *d* = 0.64), IsoPull120 PF (*p* = 0.248, *d* = 1.06); moderate effect sizes for change in IsoSqT90 RFD90 (*p* = 0.605, *d* = 0.76), IsoSqT120 RFD90 (*p* = 0.442, *d* = 0.61), IsoPress90 PF (*p* = 0.758, *d* = 0.65) and IsoPull90 PF (*p* = 0.481, *d* = 0.60); and small effect sizes for change in IsoPress90 RFD90 (*p* = 0.348, *d* = 0.20), IsoPress120 PF (*p* = 0.356, *d* = 0.28) and IsoPull90 RFD100 (*p* = 0.215, *d* = 0.34).

### 3.5. Correlation Analysis

Significant and large associations were observed between change in 200mTT mean power and IsoSqT90 PF (*r* = 0.51, *p* = 0.022), and change in 200mTT mean power and IsoPull120 PF (*r* = 0.52, *p* = 0.018) (Appendix A, Table A2). There was no significant correlation between change in 200mTT mean power and other isometric strength measures (0.01 ≤ *r* ≤ 0.34, 0.144 ≤ *p* ≤ 0.956).

## 4. Discussion

The current findings showed that the inclusion of IST resulted in a larger effect for the improvement of 200mTT mean power and all isometric strength measures than TRAD. The greater improvement in both sports-specific performances in the IST group was in corroboration with a previous finding that reported greater improvement in cycling performance in elite sprint cyclists who included IST in their training [13]. In addition, there was an association between improvement in 200mTT mean power with improved lower limb pushing and upper limb pulling strength.

Previous studies have reported that IST is superior in improving joint angle-specific force generation ability than other modes of strength training [8,14]. Therefore, when IST was performed at the joint angle that corresponded to the initiation of concentric phase of an exercise, it resulted in greater force generation at that specific joint angle [10]. Although the current study did not adopt a kayaking position during IST, the hip, knee and elbow positions adopted while performing IsoSqT and, IPB and IsoPull, respectively, were similar to the joint angle adopted during the initiation of pulling phase of the kayak stroke [5]. This probably led to the ability to generate a greater amount of force and greater ability to overcome inertial during kayaking on the ergometer.

Another possible reason for the greater improvement in IST could be because of the diminishing return of training after having performed a certain type of training for extended period of time [23]. In the current study, all the participants performed the same training program as TRAD prior to the commencement of the intervention. The smaller magnitude in strength improvement in TRAD was possibly due to the lack of change in training stimulus. In the case of the IST group, the inclusion of IST acted as a form of variation in training that resulted in greater strength adaptation [24].

In concordance with the significantly greater improvement in IsoSqT90 PF, and larger effect in the improvement of 200mTT and IsoPull120 PF in favor of IST, the results also showed significantly large associations between improvement in 200mTT mean power and improvement in PF achieved from IsoSqT90 and IsoPull120. Similarly, a previous study also reported significant correlation between PF achieved from IsoSqT90 and IsoPull120 with both 200-m ergometer kayaking mean power and on-water kayaking time [5]. A possible reason for these observations could be because the pulling phase of the kayak stroke is the result of a summation of forces that is initiated by simultaneous hip extension, knee extension and ankle plantar flexion [25]. This explains why there was a significant correlation between the improvement in 200mTT mean power and improvement in PF achieved from IsoSqT90. In addition, during the pulling phase, the shoulder joint also performs a forceful extension and internal rotation action by increasing the activation of the latissimus dorsi muscles [26]. Similarly, latissimus dorsi activity is high while performing IsoPull [27]. This explains why there was a significant correlation between the improvement in 200mTT mean power and improvement in PF achieved from IsoPull120. Overall, these findings suggest that the improvement in lower limb strength and upper limb pulling strength may be integral to the improvement in kayaking performance.

This study has some limitations that should be acknowledged. Firstly, the magnitude of strength gain while performing TRAD and IST is influenced by the amount and rate of force produced during each repetition [10,28,29,30]. Therefore, participants’ compliance to perform each repetition with maximal effort would greatly affect the magnitude of strength gain. As force and rate of force development were not measured during training, these factors could have resulted in the individual differences observed in the current findings. Secondly, the current results only reflect the outcome of a six-week training period. The long-term effects of including IST into a training program are still not known. Thirdly, although the total number of sets for all exercises were the same, the intensity and time under tension were not equated. While IST was performed at maximal intensity, TRAD was performed at submaximal intensity. This could be a reason for the greater improvement observed in IST. Finally, IST resulted in improved 200mTT mean power, which has been shown to be highly correlated to 200-m on-water sprint time, actual on-water performance was not assessed and would require further investigation.

## 5. Conclusions

The current study showed that the inclusion of IST resulted in greater improvement in both sprint kayaking and strength performance as compared to TRAD. One possible reason for the current finding was possibly because IST was performed at joint positions similar to those adopted during the initiation of pulling phase of the kayak stroke, resulting in enhanced ability to overcome inertial and/or drag force during kayaking. Another reason could be because the inclusion of IST acted as a form of variation in training that resulted in greater strength adaptation. Together with the significant association between improvement in sprint kayaking performance with the improvement of lower limb strength and upper limb pulling strength, the greater effect in strength improvement observed in IST suggests that strength increment was one of the reasons contributing to enhance mean paddling power.

## Figures and Tables

**Table 1:** Strength training program.

Exercises	Sets × Repetitions					
TRAD	Week 1	Week 2	Week 3	Week 4	Week5	Week 6
Back Squat	4 × 8	4 × 8	4 × 6	4 × 6	4 × 4	2 × 4
Bench pull	4 × 8	4 × 8	4 × 6	4 × 6	4 × 4	2 × 4
Bench Press	4 × 8	4 × 8	4 × 6	4 × 6	4 × 4	2 × 4
Weighted pull up	4 × 8	4 × 8	4 × 6	4 × 6	4 × 4	2 × 4
Dumbbell shoulder press	4 × 8	4 × 8	4 × 6	4 × 6	4 × 4	2 × 4
Single arm seated row	4 × 8	4 × 8	4 × 6	4 × 6	4 × 4	2 × 4
IST						
Back Squat	2 × 8	2 × 8	2 × 6	2 × 6	2 × 4	1 × 4
Isometric squat at 90° knee angle	2 × 5	2 × 5	2 × 5	2 × 5	2 × 5	1 × 5
Bench pull	2 × 8	2 × 8	2 × 6	2 × 6	2 × 4	1 × 4
Isometric bench pull at 120° elbow angle	2 × 5	2 × 5	2 × 5	2 × 5	2 × 5	1 × 5
Bench Press	2 × 8	2 × 8	2 × 6	2 × 6	2 × 4	1 × 4
Isometric bench press at 90° elbow angle	2 × 5	2 × 5	2 × 5	2 × 5	2 × 5	1 × 5
Weighted pull up	4 × 8	4 × 8	4 × 6	4 × 6	4 × 4	2 × 4
Dumbbell shoulder press	4 × 8	4 × 8	4 × 6	4 × 6	4 × 4	2 × 4
Single arm seated row	4 × 8	4 × 8	4 × 6	4 × 6	4 × 4	2 × 4

**Table 2:** Analysis of 200-m kayak ergometer mean power and isometric peak force.

		Mean Power (W)	IsoSqT 90 PF (N)	IsoSqT 120 PF (N)	IsoPress90 PF (N)	IsoPress 120 PF (N)	IsoPull 90 PF (N)	IsoPull 120 PF (N)
TRD	Pre	220.8 (71.0)	1699.1 (442.7)	2471.4 (848.3)	662.4 (198.9)	1071.3 (260.8)	644.2 (177.9)	906.4 (242.0)
	Post	233.6 (71.6)	1737.2 (432.6)	2552.7 (831.7)	722.2 (225.9)	1245.7 (254.4)	704.9 (221.2)	986.7 (254.9)
	95% CI	−25.2; −1.8	−156.4; 80.2	−214.6; 52.0	−193.8; 74.2	−257.4; −91.4	−134.1; 12.7	−146.0; −14.6
	*p*	0.028	0.485	0.201	0.339	0.001	0.094	0.022
	d	0.18	0.09	0.09	0.28	0.68	0.30	0.32
IST	Pre	223.7 (66.9)	1974.8 (289.9)	2758.8 (421.0)	710.3 (247.3)	1213.2 (286.3)	680.7 (120.0)	1023.3 (238.1)
	Post	254.7 (69.8)	2201.2 (302.0)	3007.0 (610.3)	916.4 (286.9)	1484.4 (446.0)	807.0 (155.6)	1218.9 (273.8)
	95% CI	−42.6; −19.5	−276.2; −176.6	−540.9; 44.3	−327.7; −84.5	−497.1; −45.3	−210.3; −42.3	−246.1; −145.1
	*p*	<0.001	<0.001	0.087	0.004	0.024	0.008	<0.001
	d	0.45	0.76	1.04	0.77	0.72	0.90	0.76
Time × Group Interaction	F	5.81	11.02	1.38	11.047	0.828	1.77	9.91
	*p*	0.027	0.004	0.255	0.004	0.375	0.200	0.006
	η^2^ *_p_*	0.24	0.38	0.07	0.38	0.04	0.09	0.36
Time Main Effect	F	37.50	21.74	5.38	11.05	17.55	14.39	56.73
	*p*	<0.001	<0.001	0.032	0.004	0.001	0.001	<0.001
	η^2^ *_p_*	0.68	0.55	0.23	0.16	0.49	0.44	0.76
Group Main Effect	F	0.16	5.04	1.48	1.45	2.029	0.90	2.45
	*p*	0.695	0.037	0.24	0.244	0.171	0.356	0.135
	η^2^ *_p_*	0.01	0.22	0.08	0.075	0.10	0.05	0.12

IsoPress90 = isometric bench press at 90° elbow angle, IsoPress120 = isometric bench press at 120° elbow angle, IsoPull90 = isometric bench pull at 90° elbow angle, IsoPull120 = isometric bench pull at 120° elbow angle, IsoSqT90 = isometric squat at 90° knee angle, IsoSqT120 = isometric squat at 120° knee angle, PF = peak force.

**Table 3:** Analysis of isometric rate of force development.

		IsoSqT90 RFD90 (N·s^−1^)	IsoSqT120 RFD90 (N·s^−1^)	IsoPress90 RFD90 (N·s^−1^)	IsoPress120 RFD90 (N·s^−1^)	IsoPull90 RFD90 (N·s^−1^)	IsoPull120 RFD90 (N·s^−1^)
TRAD	Pre	2692.3 (1689.1)	3048.4 (1844.2)	3017.9 (1821.0)	4557.0 (2282.0)	3799.5 (1184.7)	3655.4 (2533.4)
	Post	2586.9 (891.6)	2813.8 (1027.2)	3967.0 (2299.5)	5487.5 (2615.7)	4275.9 (1274.4)	4456.0 (2291.2)
	95% CI	−838.4; 1049.2	−920.0; 1389.2	−1890.1; 391.9	−1852.2; −8.79	−1523.3; 570.5	−1706.4; 105.2
	*p*	0.806	0.657	0.172	0.048	0.330	0.077
	d	0.08	0.16	0.46	0.38	0.39	0.33
IST	Pre	3067.8 (1509.0)	4962.5 (2403.6)	4054.3 (2339.8)	6242.6 (2548.1)	4656.0 (1999.9)	6856.7 (2934.6)
	Post	4620.0 (2139.6)	6296.8 (2298.1)	5695.9 (2733.2)	8134.9 (3129.4)	6045.7 (2139.9)	9171.6 (2816.9)
	95% CI	−2550.1; −554.3	−1988.6; −680.0	−2654.8; −628.4	−3160.2; −624.4	−2131.2; −648.2	−3139.4; −1490.4
	*p*	0.007	<0.001	0.005	0.008	0.002	<0.001
	d	0.84	0.57	0.65	0.66	0.67	0.80
Time × Group Interaction	F	7.45	0.715	1.59	1.93	2.59	7.82
	*p*	0.14	0.015	0.224	0.182	0.125	0.012
	η^2^*_p_*	0.29	0.28	0.08	0.10	0.13	0.30
Time Main Effect	F	5.68	3.51	0.03	16.60	10.83	33.11
	*p*	0.028	0.077	0.867	0.001	0.004	<0.001
	η^2^*_p_*	0.24	0.16	0.002	0.48	0.38	0.65
Group Main Effect	F	3.35	10.56	4.27	3.62	3.45	11.72
	*p*	0.084	0.004	0.053	0.073	0.080	0.003
	η^2^*_p_*	0.16	0.37	0.19	0.17	0.16	0.39

IsoPress90 = isometric bench press at 90° elbow angle, IsoPress120 = isometric bench press at 120° elbow angle, IsoPull90 = isometric bench pull at 90° elbow angle, IsoPull120 = isometric bench pull at 120° elbow angle, IsoSqT90 = isometric squat at 90° knee angle, IsoSqT120 = isometric squat at 120° knee angle, RFD90 = rate of force development (0–90 ms).

## Data Availability

The data presented in this study are available on request from the corresponding author. The data are not publicly available as it has not been approved by the Institutional Review Board.

## Appendix A

**Table A1:** Reliability data for 200-m kayak ergometer mean power and isometric strength measures.

	ICC	95%CI	%TE
200mTT	0.99	0.98–0.99	3.0
IsoSqT90 Peak Force	0.93	0.88–0.95	5.1
IsoSqT90 RFD90	0.84	0.73–0.88	8.9
IsoSqT120 Peak Force	0.95	0.90–0.97	4.9
IsoSqT120 RFD90	0.87	0.72–0.88	8.5
IsoPress90 Peak Force	0.99	0.97–0.99	3.6
IsoPress90 RFD90	0.97	0.93–0.98	7.6
IsoPress120 Peak Force	0.97	0.94–0.98	4.0
IsoPress120 RFD90	0.97	0.90–0.98	7.4
IsoPull90 Peak Force	0.98	0.93–0.99	3.4
IsoPull90 RFD90	0.97	0.90–0.98	7.9
IsoPull120 Peak Force	0.99	0.98–0.99	3.8
IsoPull120 RFD90	0.91	0.88–0.93	7.6

IsoPress90 = isometric bench press at 90° elbow angle, IsoPress120 = isometric bench press at 120° elbow angle, IsoPull90 = isometric bench pull at 90° elbow angle, IsoPull120 = isometric bench pull at 120° elbow angle, IsoSqT90 = isometric squat at 90° knee angle, IsoSqT120 = isometric squat at 120° knee angle, 200mTT = 200-m kayak ergometer mean power, RFD90 = rate of force development at 0–90 ms, %TE = relative typical error.

**Table A2:** Correlation between 200-m kayak ergometer mean power and isometric strength measures.

	IsoSqT90 PF	IsoSqT90 RFD90	IsoSqT120 PF	IsoSqT120 RFD90	IsoPress90 PF	IsoPress90 RFD90	IsoPress120 PF	IsoPress120 RFD90	IsoPull 90 PF	IsoPull 90 RFD90	Isopull 120 PF	IsoPull 120 RFD90
Mean Power	0.509 *	0.246	0.340	0.117	0.333	0.339	0.013	0.152	0.325	0.316	0.522 *	0.283
95%CI	0.09; 0.78	−0.22; 0.62	−0.12; 0.68	−0.34; 0.53	−0.13; 0.68	−0.12; 0.68	−0.43; 0.45	−0.31; 0.56	−0.14; 0.67	−0.142; 0.67	010; 0.78	−0.18; 0.64
*p*	0.022	0.296	0.143	0.622	0.152	0.144	0.956	0.521	0.161	0.174	0.018	0.227

IsoPress90 = isometric bench press at 90° elbow angle, IsoPress120 = isometric bench press at 120° elbow angle, IsoPull90 = isometric bench pull at 90° elbow angle, IsoPull120 = isometric bench pull at 120° elbow angle, IsoSqT90 = isometric squat at 90° knee angle, IsoSqT120 = isometric squat at 120° knee angle, RFD90 = rate of force development (0–90 ms). * Denotes significant correlation (*p* < 0.05).
